# Histological analysis of saphenous veins surgically harvested by with or without electrocautery

**DOI:** 10.1186/1749-8090-10-S1-A185

**Published:** 2015-12-16

**Authors:** Rui Almeida, Daniel Almeida, Rodrigo Freitas, Carlos Floriano

**Affiliations:** 1 Faculdade Assis Gurgacz,Cascavel, Paraná, Brazil; 2 Parana Western State University, Cascavel, Paraná, Brazil; 3 ICCOP, Cascavel, Paraná, Brazil

## Background/Introduction

The use of electrocauterization in the harvesting of the saphenous vein, to be used as grafts in coronary artery bypass grafting (CABG) is a common practice in almost all units of cardiovascular surgery.

## Aims/Objectives

To evaluate, if the use of electrocauterization, in the harvesting of the saphenous vein has a deleterious effect, in the histological aspect of the vein, and if it correlates to the clinical result.

## Method

Twenty-four patients underwent CABG and in twelve of them electrocautery was used (Group 1) to harvest the saphenous vein and in another twelve it was not (Group 2). Vein segments were sent for analysis after fixed in 10% formalin for 24 hours. These segments were vertically cut and two fragments were removed for histological analysis. They were stained with hematoxylin and eosin according to the normal protocol. All samples were analyzed by a blinded observer, which looked for morphological changes, especially acute ones in the endothelium, muscle tunics and adventitia. The acute changes investigated were: necrosis, degeneration, inflammation, thrombosis, structural rupture signs and the presence of hemorrhage. The mid-term results of the grafts were observed clinically.

## Results

In both the groups there was no change in the endothelium or limiting membrane. In three cases (25%) of Group 1 there was the appearance of pyknotic nuclei, likely exaggerated by cauterization. In one case (8.33%), the Group 2 had a recent hemorrhage adventitia. The blind observer only managed to identify a use case cautery one sample, in Group 1, apart from those who had pyknotic nuclei.

## Discussion/Conclusion

Despite the small sample, collected, it was possible to identify four cases where cauterization was used for the dissection of the saphenous vein. There was no correlation of the same with the clinical course of patients.

